# Environmental cleaning to prevent COVID-19 infection. A rapid systematic review

**DOI:** 10.1590/1516-3180.2020.0417.09092020

**Published:** 2020-11-13

**Authors:** Patrícia Mitsue Saruhashi Shimabukuro, Márcio Luís Duarte, Aline Mizusaki Imoto, Álvaro Nagib Atallah, Eduardo Signorini Bicas Franco, Maria Stella Peccin, Mônica Taminato

**Affiliations:** I Nurse, Hospital Sancta Maggiore São Paulo, São Paulo (SP), Brazil; Master's Student, Master's Program, Escola Paulista de Enfermagem, Universidade Federal de Sao Paulo (UNIFESP), Sao Paulo (SP), Brazil.; II MD, MSc. Musculoskeletal Radiologist, Webimagem, São Paulo (SP), Brazil; Evidence-Based Health Program, Universidade Federal de São Paulo (UNIFESP), São Paulo (SP), Brazil.; III PT, PhD. Professor, Professional and Academic Master's Program in Health Sciences, Evidence-Based Health Laboratory, Escola Superior de Ciências da Saúde (ESCS), São Paulo (SP), Brazil.; IV MD, PhD. Head of Department of Evidence-Based Health, Universidade Federal de São Paulo (UNIFESP), São Paulo (SP), Brazil.; V PT, MSc. Doctoral student, Department of Evidence-Based Health, Universidade Federal de São Paulo (UNIFESP), São Paulo (SP), Brazil.; VI PT, PhD. Associate Professor, Department of Human Movement Sciences, and Advisor of Evidence-Based Health Program, Universidade Federal de São Paulo (UNIFESP), São Paulo (SP), Brazil.; VII PhD. Nurse, Escola Paulista de Enfermagem (EPE), Universidade Federal de São Paulo (UNIFESP), São Paulo (SP), Brazil.

**Keywords:** COVID-19 [supplementary concept], Coronavirus infections, Environmental monitoring, Disinfection, Sterilization, SARS-CoV-2, Ambiance cleaning, Ambiance hygiene, Environmental cleaning, Environment hygiene, Cleaning

## Abstract

**BACKGROUND:**

Faced with a pandemic, all healthcare actions need to reflect best practices, in order to avoid high transmissibility, complications and even hospitalizations. For hospital environments, the products recommended and authorized by regulatory institutions for environmental cleaning and disinfection need to be highly effective.

**OBJECTIVE:**

To identify, systematically evaluate and summarize the best available scientific evidence on environmental cleaning to prevent COVID-19 infection.

**DESIGN AND SETTING:**

A systematic review of studies analyzing cleaning products that inactivate coronavirus, conducted within the evidence-based health program of a federal university in São Paulo (SP), Brazil.

**METHODS:**

A systematic search of the relevant literature was conducted in the PubMed, EMBASE, Cochrane Library, CINAHL and LILACS databases, for articles published up to May 27, 2020, relating to studies evaluating cleaning products that inactivate coronavirus in the environment.

**RESULTS:**

Seven studies were selected. These analyzed use of 70% alcohol, detergent, detergent containing iodine, household bleach, sodium hypochlorite, hydrogen peroxide, chlorine dioxide, glutaraldehyde, ultraviolet irradiation and plasma air purifier. The effectiveness of treating sewage with sodium hypochlorite and chlorine dioxide was also evaluated.

**CONCLUSION:**

Disinfection of environments, especially those in ordinary use, such as bathrooms, needs to be done constantly. Viral inactivation was achieved using chlorine-based disinfectants, alcohol, detergents, glutaraldehyde, iodine-containing detergents, hydrogen peroxide compounds and household bleaches. Alcohol showed efficient immediate activity. In sewage, sodium hypochlorite had better action than chlorine dioxide.

**REGISTRATION NUMBER:**

DOI: 10.17605/OSF.IO/YC5P4 in the Open Science Framework.

## INTRODUCTION

Severe acute respiratory syndrome coronavirus 2 (SARS-CoV-2), which is an emerging respiratory pathogen, causes the COVID-19 disease. Some issues regarding its main epidemiological, clinical and virological characteristics, and particularly its capacity for dissemination, are being discovered. Evidence from other coronavirus diseases, for example severe acute respiratory syndrome (SARS) and Middle East respiratory syndrome (MERS), and the experiences from control and prevention of COVID-19 adopted so far, suggests that it is transmitted through droplets and contact. Thus, COVID-19 can be spread through aerosols relating to procedures that produce aerosolization, such as swab sample collection, intubation and aspiration, among others.^1^^,^^2^

Prevention and control measures for the new coronavirus need to include hand hygiene, disinfection of surfaces (notably those that are very frequently touched), respiratory etiquette, avoidance of touching one's face and use of masks. When all these measures are combined, they are efficient for prevention of human-human transmission of COVID-19.^3^^,^^4^ With the emergence of SARS caused by the new coronavirus, the world has seen the consequences of respiratory transmission between people. It acknowledges that the incubation period is 2 to 10 days, which facilitates its propagation on inanimate surfaces.^4^^,^^5^

Information relating to specific inactivation of COVID-19 has recently emerged. Current studies demonstrate that for human coronaviruses to be inactivated (for example SARS coronavirus, MERS coronavirus or endemic human coronavirus (HCoV)), use of products such as ethanol, hydrogen peroxide or sodium hypochlorite, in addition to other biocidal agents used in chemical disinfection, like benzalkonium chloride or chlorhexidine digluconate, is effective.^5^^,^^6^ Therefore, early containment and prevention of further spread will be crucial in order to stop the ongoing outbreak and control this new infectious disease.

The presence and persistence of COVID-19 in clinical settings and on surfaces are being extensively researched. Experiments performed under controlled laboratory conditions have provided some indications of the ability of the virus to survive under different environmental conditions. This transmission can develop if there is inadequate waste management and inappropriate handling of personal protective equipment (PPE) in developing countries.^5^

Research conducted specifically on COVID-19 has indicated that the new coronavirus may survive for at least 72 hours, and that it is more stable on the plastic or stainless steel substrates commonly found in operating rooms.^5^^–^^7^ Persistence of the virus in the environment is known to be a means for transmission of infection. Contact with contaminated fomites is one of the pathways involved in spreading the infection of SARS-CoV-2.^7^ The virus is most frequently transmitted through inhalation of respiratory droplets or their deposition in the mucosa (mouth, nose and eyes).^8^

Faced with a pandemic, all healthcare actions need to reflect best practices, in order to avoid high transmissibility, complications and even hospitalizations. For hospital environments, the products recommended and authorized by regulatory bodies for environmental cleaning and disinfection need to be highly effective. The Centers for Disease Control (CDC) have published guidelines for patients with suspected or actual infection with SARS-CoV-2 who are seen at healthcare services. The guidelines mention the importance of having a protocol to guide the team for cleaning the environment and equipment.^9^^,^^10^

Within this scenario, it can also be highlighted that it is important to draw up protocols for a gradual return to everyday activities in order to ease social distancing.

## OBJECTIVES

The aim of this study was to identify, systematically evaluate and summarize the best available scientific evidence on environmental cleaning to prevent COVID-19 infection.

## METHODS

### Study model

This study was a rapid systematic review. The research protocol was registered in the Open Science Framework.

### Inclusion criteria

The search was performed in accordance with the Preferred Reporting Items for Systematic Reviews and Meta-Analyses (PRISMA) guidelines. Given the limited number of studies on environmental cleaning to prevent COVID-19 infection that might have been published so far, the purpose of this review was to map the knowledge that currently existed on this subject and identify the designs of these studies according to their level of evidence. There was no restriction in relation to origin, language or publication status of the study.

### Phenomena of interest

The phenomena of interest for this review comprised cleaning practices performed in healthcare services with the aim of cleaning environments that had possibly become contaminated with suspected or confirmed COVID-19 infection.

### Type of intervention

Use of products recommended and authorized by regulatory bodies that presented safety and efficiency with regard to cleaning the environment comprised the intervention.

### Type of outcomes

The outcomes selected were effectiveness of disinfection, use of products for cleaning the environment and elimination of environmental contamination.

### Selection of studies and data extraction

Identification of eligible studies followed a two-stage process accomplished by two independent reviewers. Any disagreement was resolved by reaching a consensus. In the first stage, after exclusion of duplications, the titles and abstracts of the references identified through the search strategy were evaluated and the potentially eligible studies were pre-selected. In the second stage, a full-text evaluation of the pre-selected studies was carried out to confirm their eligibility. The selection process was performed through the Rayyan platform (https://rayyan.qcri.org).^11^

### Research methods for selecting studies

The search strategy was elaborated in accordance with the following research question: Is there any evidence that it is important to use cleaning and disinfection products against SARS-CoV-2?

The searches were elaborated using health science descriptors and adapted for use in each of the databases selected: Cochrane Library (Wiley); Embase (Elsevier); VHL Portal; Medical Literature Analysis and Retrieval System Online (MEDLINE, PubMed); CINAHL; Web of Science; Scopus; and Opengrey (https://opengrey.eu). These descriptors were as follows: “severe acute respiratory syndrome coronavirus 2”[Supplementary Concept] OR “severe acute respiratory syndrome coronavirus 2”[All Fields] OR “sars cov 2”[All Fields]) AND (“environment”[MeSH Terms] OR “environment”[All Fields]) AND (“disinfection”[MeSH Terms] OR “disinfection”[All Fields]).

A manual search was conducted in the references of the primary and secondary studies that were identified through the electronic search. The search strategies developed and used for each electronic database were performed between April 29, 2020 and May 27, 2020. They are presented in Table 1. There were no restrictions on languages or forms of publication.

**Table 1:** Search strategy according to the corresponding databases

Database	Search strategy
Cochrane Library	#1 MeSH descriptor: [SARS Virus] explode all trees #2 MeSH descriptor: [Coronavirus Infections] explode all trees #3 MeSH descriptor: [Environmental Monitoring] explode all trees #4 MeSH descriptor: [Housekeeping] explode all trees #5 MeSH descriptor: [Housekeeping, Hospital] explode all trees #6: #1 OR #2 AND #3 OR #4 OR #5
MEDLINE	#1: “Coronavirus Infections”[MeSH] OR (Coronavirus Infection) OR (Infection, Coronavirus) OR (Infections, Coronavirus) OR (Middle East Respiratory Syndrome) OR (MERS (Middle East Respiratory Syndrome)) OR “COVID-19 [Supplementary Concept]”[MeSH] OR (2019 novel coronavirus infection) OR (COVID19) OR (coronavirus disease 2019) OR (coronavirus disease-19) OR (2019-nCoV disease) OR (2019 novel coronavirus disease) OR (2019-nCoV infection) OR “SARS Virus”[MeSH] OR (Severe Acute Respiratory Syndrome Virus) OR (SARS-Related Coronavirus) OR (Coronavirus, SARS-Related) OR (SARS Related Coronavirus) OR (SARS-CoV) OR (Urbani SARS-Associated Coronavirus) OR (Coronavirus, Urbani SARS-Associated) OR (SARS-Associated Coronavirus, Urbani) OR (Urbani SARS Associated Coronavirus) OR (SARS Coronavirus) OR (Coronavirus, SARS) OR (Severe acute respiratory syndrome-related coronavirus) OR (Severe acute respiratory syndrome related coronavirus) OR (SARS-Associated Coronavirus) OR (Coronavirus, SARS-Associated) OR (SARS Associated Coronavirus) #2: “Environmental Monitoring”[MeSH] OR (Monitoring, Environmental) OR (Environmental Surveillance) OR (Surveillance, Environmental) OR “Housekeeping”[MeSH] OR (Housework) OR “Housekeeping, Hospital”[MeSH] OR (Hospital Housekeeping) OR (Hospital Housekeepings) OR (Housekeepings, Hospital) #3: #1 AND #2
EMBASE (OvidSP)	#1: ‘covid 19’/exp OR ‘SARS coronavirus’/exp OR ‘Coronavirus infection’/exp #2: Environmental Monitoring/ exp or Housekeeping/ exp or Hospital service/ exp #3: #1 AND #2
LILACS	#1: “Vírus da Sars” or (Virus del SRAS) or (SARS Virus) or (CoV-SARS) or (CoV-SRAG) or (Coronavirus Associado a SARS) or (Coronavirus Relacionado à Síndrome Respiratória Aguda Grave) or (SARS-CoV) or (SRAG-CoV) or (Vírus SARS) or (Vírus da Pneumonia Asiática) or (Vírus da Síndrome Respiratória Aguda Grave) or (Vírus da Síndrome Respiratória Aguda Severa) or (mh:B04.820.504.540.150.113.937$) #2: “Monitoramento Ambiental” or (Monitoreo del Ambiente) or (Environmental Monitoring) or (Combate à Poluição) or (Controle Ambiental) or (Controle da Contaminação Ambiental) or (Monitoramento Ecológico) or (Prevenção da Poluição) or (Redução da Poluição) or (mh:N06.850.460.350.080$) or (mh:N06.850.780.375$) or (mh:SP2.001.030.040$) or (mh:SP2.036.010.008$) or (mh:SP4.102.072.092.693.364$) or (mh:SP4.102.072.573.954$) or (mh:SP4.127.413.629.885$) or (mh:SP5.006.067.100.150$) or (mh:SP8.473.654.412.052.005.030.050.010$) or (mh:VS4.001.001$) or “Serviço de Limpeza” or (Servicio de Limpieza) or (Housekeeping) or (Limpeza) or (mh:N02.508$) or “Serviço Hospitalar de Limpeza” or (Servicio de Limpieza en Hospital) or (Housekeeping, Hospital) or (mh:N02.278.216.500.968.412$) or (mh:N02.508.472$) or (mh:N04.452.442.452.422.412$) or (mh:VS3.002.001.001.011.001$) #3 #1 AND #2
CINAHL	#1: (Sars virus) OR (Coronavirus infections) OR (covid-19 or coronavirus or 2019-ncov) #2: (environmental monitoring) OR (Housekeeping) OR (Housekeeping, Hospital) #3: #1 AND #2

## RESULTS

### Studies selected

**Figure 1:**
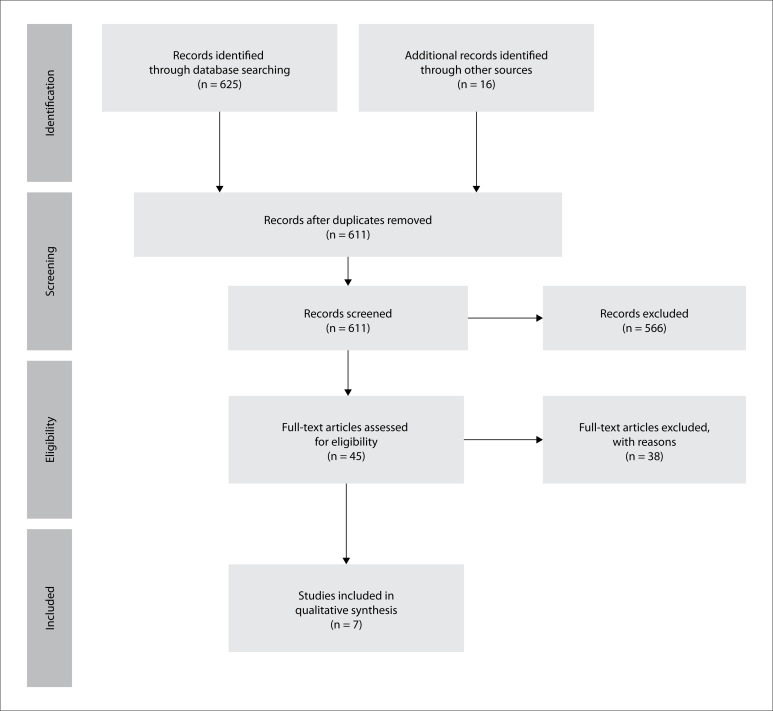
Preferred Reporting Items for Systematic Reviews and Meta-Analyses (PRISMA) flow diagram for study selection.

The systematic review yielded 641 papers; 30 of them were duplicates. After the titles and abstracts had been read by two independent evaluators through the Rayyan online platform, 45 articles were included for the full text to be read. Through this, seven studies were included. The Preferred Reporting Items for Systematic Reviews and Meta-Analyses (PRISMA) flowchart is shown in Figure 1. The years of publication ranged from 2000 to 2020. These studies were conducted in Italy, Canada, Peru, Australia, Germany and China (two studies). The details of the seven studies selected are shown in Table 2.^7^^,^^12^^–^^17^

**Table 2:** Analysis on the articles included in the study

Study	Study design	Environment/ surface studied	Cleaning and disinfection methods/products	Results and conclusion
Sizun et al.^16^	Laboratory	Aluminum, sterile latex surgical gloves and sterile sponges.	Several common disinfectant agents were evaluated: 70% (v/v) ethanol; detergent containing 0.75% free iodine; 1.5% freshly prepared (v/v) household bleach; and soap. 12 aluminum pieces of 1 cm in diameter were washed with tap water and disinfected with 70% ethanol for 30 minutes, followed by further disinfection through heating.	The free iodine detergent reduced the degree of infection of the virus by at least 50%. HCoV-OC43 was more sensitive to free iodine detergent than was HCoV-229E, since it was neutralized with a lower concentration of this chemical disinfectant. Use of soap and ethanol is believed to be effective, since alcohol and detergents destabilize the lipid bilayer of viruses.
Booth et al.^15^	Laboratory	Hospital environment. Environmental samples were collected from 19 inpatient rooms for patients infected with SARS.	Air samples and patient room surfaces, handrails, telephones, televisions, remote controls, switches, medical records, beds, bathroom furniture and utensils, corridors adjacent to rooms, hand sanitizing stations, personal protective equipment and nursing rooms. The cleaning protocol of the hospitals included consisted of cleaning and disinfection of surfaces, equipment and floors with products based on hydrogen peroxide. Detection of SARS-CoV by means of RT-PCR.	The study data showed that an environmental cleaning protocol with well-designed routines was effective. The environments were disinfected twice a day, as also were the frequently touched surfaces. In this study, only two surfaces were positive: the refrigerator at the nursing station and the television remote control in the patients’ rooms.
Lai et al.^12^	Laboratory	Examination request paper, waterproof disposable cloak and non-disposable cotton cloak.	Sodium hypochlorite, household detergent and a hydrogen peroxide compound.	The risk of infection from contact with contaminated droplets on paper was small. The three products reduced the viral load after five minutes of incubation. Regarding the type of cloak, those made with higher-absorption material such as cotton were preferable to those made of non-absorbent materials. The virus was easily inactivated using common disinfectants.
Rabenau et al.^7^	Case control	Hospital.	Test of the eight products most used in Germany, with exposures of 30 seconds, 30 minutes and 60 minutes to evaluate the effectiveness of SARS. The products used were aldehyde, formaldehyde, active oxygen and aldehyde for instruments and alcohol for hand hygiene. Evaluations were done with minimum organic load reduction factors (RFs): 0.3% serum albumin (BSA), 10% fetal calf serum (FCS) and 0.3% BSA with 0.3% sheep erythrocytes.	All were effective for SARS-CoV inactivation.
Wang et al.^14^	Laboratory	Feces, urine and water.	Sodium hypochlorite and chlorine dioxide. Detection of SARS-CoV by means of RT-PCR.	Free chlorine inactivated SARS-CoV better than chlorine dioxide. Free residual chlorine over 0.5 mg/L for chlorine or 2.19 mg/L for chlorine dioxide in wastewater ensured complete inactivation of SARS-CoV. The product concentration was inverse to the time taken for SARS-CoV inactivation.
Walker et al.^17^	Laboratory	Experimental chamber.	250 nm ultraviolet irradiation. Viruses were aerosolized in the experiment and were found to be susceptible to UV radiation, with significant reductions in viral load. The coronavirus demonstrated high sensitivity: only 12% of the virus survived exposure to 599 μW-s/cm^2^ of UV-C.	Air disinfection through an association of HEPA filter with 254 nm UV-C can be an effective tool for inactivating viral aerosols. Among the viruses examined, adenovirus was the most resistant to 254 nm UV-C and needed to be exposed to high doses of UV for complete inactivation.
Wang et al.^13^	Laboratory	Hospital inpatient areas used by contaminated patients.	Plasma air purifies the environment. Disinfection is achieved through using a tissue moistened with chlorine on surfaces that have been touched by patients affected by SARS-CoV-2 and by professionals who treated these patients. The environment samples were collected four hours after cleaning and were evaluated by means of RT-PCR.	The importance of hand hygiene and cleaning the environment to prevent the transmission of SARS-CoV-2 was shown.

v/v = volume per volume; HCoV = human coronavirus; SARS = severe acute respiratory syndrome; SARS-CoV = severe acute respiratory syndrome coronavirus; RT-PCR = reverse-transcription polymerase chain reaction; nm = nanometers; UV = ultraviolet; UV-C = ultraviolet-C; HEPA = high-effciency particulate arrestance.

### Characteristics of the studies included

One case-control study and one experimental study were carried out in hospital settings. Another five studies were laboratory tests.

### Products analyzed

#### Sodium hypochlorite at dilutions of 0.1 to 0.5%

Lai et al.^12^ analyzed paper samples, waterproof disposable cloaks and fabric cloak (cotton). Sodium hypochlorite significantly reduced the viral load after five minutes of incubation.

In the study by Wang J et al.,^13^ tissues impregnated with hypochlorite were used for cleaning and disinfecting surfaces in hospitals and personal protective equipment (PPE). Inpatient units with patients undergoing treatment for COVID-19 were selected. After this cleaning, the polymerase chain reaction (PCR) was performed on samples collected from the environment, and no presence of SARS-CoV-2 was observed. The PPE used by professionals who provided care in these environments also yielded the same results through PCR analysis.

In the study by Wang XW et al.,^14^ conducted in Wuhan, China, the presence of SARS-CoV was observed in wastewater samples containing feces and urine from a hospital and in domestic sewage and in tap water. The persistence of the virus between different types of water treatment (chlorinated or not chlorinated) was analyzed. It was found that the virus persisted in residual water with no chlorine treatment for up to three days; in feces for 14 days; and in urine for 17 days. Also, a difference in the persistence of SARS-CoV was observed at lower temperatures. When treatments with sodium hypochlorite and chlorine dioxide were implemented, the virus was inactivated.

#### Peroxygen compounds

Lai et al.^12^ evaluated the use of peroxygen compounds through cell cultures. They found that these compounds reduced the viral load of SARS-CoV after five minutes of incubation. The surfaces tested were paper samples, waterproof disposable cloaks and fabric cloaks (cotton).

#### Hydrogen peroxide

Booth et al.^15^ used hydrogen peroxide-based products to clean and disinfect the air and different surfaces such as handrails, telephones, televisions, remote controls, switches, charts, beds, furniture and bathroom utensils, in the bedrooms of patients infected with SARS-CoV. They also applied these products in corridors adjacent to these rooms, at hand sanitation stations, on personal protective equipment and in nursing rooms. The environments were disinfected twice a day, in addition to the surfaces frequently touched. According to the data from this study, environmental cleaning protocols with well-designed routines are effective. It is worth noting that only two surfaces were positive in reverse-transcription polymerase chain reaction (RT-PCR) tests: the refrigerator at the nursing station and the television remote control in the patients’ rooms. These findings highlight that environmental control measures need to be applied alongside adherence to hand hygiene among all personnel.

#### Household detergents

Lai et al.^12^ found that household detergent was able to reduce the viral load of SARS-CoV after five minutes of incubation. This was observed in relation to paper samples, waterproof disposable covers and fabric covers (cotton).

#### Product combination: 70% alcohol, glutaraldehyde, iodine detergent and household bleach

Rabenau et al.^7^ studied products for surface cleaning and the exposure time needed for each product. In this study, products with an active ingredient based on alcohol, glutaraldehyde, detergent with iodine and household bleach were used. Elimination of SARS-CoV-2 from the environment was observed, independent of the length of exposure of the surface to the product.

In a study on various types of coronavirus by Sizun et al.,^16^ 12 pieces of aluminum were cleaned with running water and disinfected with 70% alcohol for 30 minutes. The results from this study suggested that presence of the virus on the surface of materials may be the main sources of hospital infections. The use of povidone-iodine and 70% alcohol showed efficacy in eliminating the virus (SARS-CoV-2, SARS and MERS-CoV) in all the environments evaluated.

#### Ultraviolet C germicide

Walker et al.^17^ conducted an experiment in which irradiation using ultraviolet (UV)-C light through a high-efficiency particulate arrestance (HEPA) filter was correlated with the effect of UV radiation alone at 250 nanometers (nm). The murine hepatitis virus (MHV) coronavirus was found to be sensitive to the action of germicidal UV at 254 nm; only 12% of the aerosolized virus survived UV exposure at a rate of 599 microwatt-seconds (μW-sec) per square centimeter (cm^2^). The viral aerosols tested showed higher susceptibility to UV than did a liquid suspension. It was concluded that disinfection of the air using 254 nm UV-C (“germicidal” ultraviolet radiation) could be an effective tool for inactivation of viral aerosols. Among the viruses examined, adenovirus was the most resistant to 254 nm UV-C, and it needed to be exposed to high doses of UV for complete inactivation.

#### Plasma for air purification

Wang J et al.^13^ investigated plasma treatment for air purification in areas where patients were hospitalized, in association with environmental care for surfaces. The samples from this environment were negative except for three samples of pre-processed sewage and one sample after disinfection and pre-processing.

## DISCUSSION

The main reason for this review, regardless of the specific characteristics of the viruses, was to investigate the possibility that human coronavirus might be transmitted indirectly. The virus remains active on different types of surfaces, and infection can arise after the virus has come into contact with human mucosal surfaces.^18^ The present study complements the guidelines for prevention and control of COVID-19, with the aim of ensuring that the best evidence is used in managing the environment. Focusing on products and techniques that are applied consistently in communities, homes, schools, markets and healthcare facilities will help prevent transmission of the virus that causes COVID-19.

For contaminated surfaces to play a role in transmission, the respiratory pathogens need to be expelled into the environment and subsequently survive on these surfaces. These pathogens then need to be transferred to hands or to other materials at a viral load that is considered to be infectious. In addition, the pathogens need to have the ability to start an infection through contact with the eyes, nose or mouth.^19^

The cleaning process consists of removing microorganisms mechanically and chemically, thereby reducing the microbial load in this environment. Therefore, undertaking cleaning in association with disinfection is essential for obtaining significant reductions in the microbial load.^20^ The disinfection process does not eliminate bacterial spores, but it does eradicate most of the microbial agents in an environment or on a surface. Sterilization is the process that destroys microbial life in an object or on a surface through heat, pressure or chemical methods.^21^^,^^22^

The best way to prevent the spread of SARS-CoV-2 in the environment is to encourage cleaning and disinfection in places and surfaces that are touched very frequently. This is usually done together with implementation of individual non-pharmacological prevention measures such as hand hygiene, avoidance of touching the face and using masks.^4^

The seven studies included in this review presented analyses on disinfection products and techniques in order to investigate coronavirus inactivation. The analyses addressed the use of 70% alcohol, detergent, detergent containing iodine, household bleach, sodium hypochlorite, hydrogen peroxide, chlorine dioxide, glutaraldehyde, ultraviolet irradiation and plasma air purifier.^7^^,^^12^^–^^17^ These studies were mostly carried out in laboratories (five studies). This makes it possible to develop more accurate tests on product action and viral inactivation. On the other hand, studies conducted in a hospital environment with an observational design make it possible to compare the techniques and effectiveness of institutional protocols.

Sizun et al.^16^ and Lai et al.^12^ analyzed the use of different materials: aluminum, sterile latex surgical gloves, gauze, paper, sterile sponges and types of aprons. They analyzed the activity of the following products in relation to the materials: detergent, detergent containing iodine, household bleach, alcohol soap, hypochlorite sodium and a compound containing hydrogen peroxide. Among all the materials tested, these products were efficient for inactivating the coronavirus. The surfaces analyzed by these authors were sufficiently diversified to demonstrate the effectiveness and action of the products described, especially given the findings of persistent capacity of coronaviruses to survive on different surfaces that have been shown in several studies.^6^^,^^12^^,^^23^

Booth et al.,^15^ Wang et al.^13^ and Rabenau et al.^7^ analyzed the most-touched surfaces, i.e. handrails, televisions, beds, furniture, bathroom utensils, remote controls and switches, among other surfaces in the patients’ rooms, along with areas relating to direct care, such as the health center. These areas have been described during the pandemic as important related sites at which SARS-CoV-2 has been detected during this period.^24^^,^^25^ The products tested were wipes containing chlorine, alcohol, glutaraldehyde and a product based on hydrogen peroxide. All of them were effective against the coronavirus. Rabenau et al.^7^ suggests that alcohol and hypochlorite should be applied to surfaces and floors. They also recommend that glutaraldehyde should be applied to equipment that is used within care, such as for disinfection of bronchoscopes.

Ong et al.^25^ analyzed the presence of SARS-CoV-2 in hospital environments before and after the cleaning process. Sixteen out of 26 samples were positive for SARS-CoV-2. Positive results were obtained from these samples in 13 (87%) of the 15 locations inside the room (including exhaust fans) and in three (60%) of the five bathroom locations (i.e. toilet, sink and door handle). In that study, there was a significant degree of environmental contamination from patients with SARS-CoV-2, through respiratory droplets and fecal leakage. This suggested that the environment was a potential means of transmission.

Viral inactivation through use of chlorine-based disinfectants, alcohol, detergents, glutaraldehyde, iodine-containing detergents, hydrogen peroxide compounds and household bleaches has been demonstrated.^7^^,^^12^^,^^15^^,^^16^ These substances are easily accessible for use in hospital environments and domestic environments. Alcohols have immediate efficient activity.^6^^,^^26^

The present review also warns about viral aerosols that might be found in environments that are commonly used, such as bathrooms and stores. Due to lack of ventilation, these environments allow the virus to remain in suspension. There is no certainty about the viral load that may influence this transmission, but it is known that the virus particles can remain suspended in the air for hours.

Liu et al. reported that the SARS-CoV-2 outbreak could be correlated with transmission through at least three means:^27^

- Inhalation of liquid droplets produced by infected people or by their contacts.
- Presence of the pathogen as aerosols in confined areas.
- Contact with surfaces contaminated with SARS-CoV-2.

In that study,^27^ research was carried out on the environment in a hospital dedicated to patients with SARS-CoV-2. The places with the greatest presence of the virus were the room in which personal protective equipment (PPE) was removed and the mobile toilet used in the hospital. In this way, the importance of frequent cleaning of the environment is evident.

Environmental control measures implemented in association with disinfection techniques such as the use of laminar flow ventilation with UV-C and plasma treatment are considered feasible.^8^^,^^13^^,^^17^^,^^27^ In an experimental study, Walker et al.^17^ showed the benefits of using UV-C light in association with a laminar flow device, for viral inactivation in the air treatment system. However, the data provided did not allow any guarantee of effectiveness in relation to different types of coronavirus and to the capacity of the technique, according to the flow and air passage of each system. Wang et al.^13^ demonstrated that use of an environmental surface cleaning protocol in association with air treatment with plasma was suitable for an environmental contamination test to screen for SARS-CoV-2.

In a study by Van Doremalen et al.,^8^ it was observed that use of laminar flow ventilation for the place where healthcare professionals remove their PPE was a favorable alternative for prevention of proliferation of the virus in the environment. A high concentration of the virus was observed in toilets and changing rooms without this type of ventilation, in their study.

It needs to be borne in mind that SARS-CoV-2 can be active on inanimate surfaces for up to nine days at temperatures of 30 °C. Therefore, environmental cleaning needs to be intensified, especially for the areas that are most touched.^5^^–^^7^ The resistance of the virus on inanimate surfaces is influenced by the following factors:^12^^,^^23^^,^^28^

- The type of surface.
- The temperature of the environment.
- The relative humidity of the air.

Surfaces like plastic show viral activity for long periods, and this can last for up to 20 days. Low temperatures and low relative humidity enable persistence of human coronaviruses for longer periods. At lower temperatures, greater stability of the virus is observed.

Regarding the persistence of the virus at different temperatures, which was much discussed at the beginning of the pandemic, Wang et al.^13^ pointed out that at lower temperatures, longer survival of this virus in the environment is observed. Hence, these authors considered that lower temperatures would be ideal for its dissemination. On the other hand, in a study conducted by Wang et al.^14^ in a public sauna of 300 square meters (m^2^) at a temperature between 25 and 41 °C and relative humidity of approximately 60%, an outbreak of SARS-CoV-2 was observed among seven patients who had used the same space within a one-week period. All the patients had clinical symptoms and positive results.

The way in which sewage is treated in different countries is also relevant. Wang et al.^13^ compared the effectiveness of treating sewage with sodium hypochlorite and chlorine dioxide as a control measure against SARS-CoV-2, to avoid water contamination. Sodium hypochlorite was observed to have better action than chlorine dioxide. Moreover, the time taken to reach virus inactivation was inversely proportional to the concentration of the product. These findings provide a warning about the importance of sewage treatment, even though there is little evidence so far to support this route as a potential means of infection. It was also found in that study that other human coronaviruses survived for about two days in dechlorinated tap water and hospital wastewater at 20 °C.

Wang et al.^14^ found that places where there was no effective sewage treatment had viral loads that potentially posed a risk of transmission of the virus. Only limited studies on waste management have been conducted but, nonetheless, evidence is emerging that viral fragments are present in untreated excrement and sewage.^14^^,^^20^ Provision of good drinking water, sanitation and hygiene conditions is essential for protecting human health in all outbreaks of infectious diseases, including COVID-19.

During the current pandemic, through fear, some people have been increasing the concentrations of cleaning products that they use at home. We would warn about the importance of not increasing the concentrations of these products and about the undesirability of making homemade preparations. Depending on the substance used, higher concentrations may give rise to chemical reactions that could cause poisoning of the person who is performing this manipulation. Studies have shown that since the beginning of the SARS-CoV-2 pandemic, there has been an increase in the number of cases of exogenous poisoning seen in urgent and emergency services in the United States.^9^^,^^10^^,^^29^

Given the public health challenges relating to social issues and the need for a gradual and programmed return to activities, we suggest that measures to improve hand hygiene, social distancing, use of masks, cleaning and environmental disinfection among the population should be considered as nonpharmacological strategies towards prevention of COVID-19.

One of the limitations of our study was that data on the effectiveness of various types of disinfection against SARS-CoV were scarce. Nonetheless, even though the outbreak of this disease is very recent, it was possible to ascertain the action and efficiency of the most usual and accessible disinfectants and products, at the concentrations and exposure times used, and to demonstrate that their activity was reproducible, even with different types of organic load.

Unfortunately, we did not find any data on certain substances and materials that are widely publicized and even commercialized, such as ozone, in the articles that we were able to assess. Moreover, it also needs to be taken into account that not all of the studies cited the time taken for the substance to have its effect, with regard to elimination of the virus.

We can highlight that the present study demonstrates that a variety of products and techniques enable efficient elimination of SARS-CoV-2 in the environment. These can be used in public, domestic and hospital environments, in a way that is accessible for the population, in terms of both management and product costs. In addition, we showed that cleaning measures implemented within the infrastructure of toilets and changing rooms are essential for preventing the spread of the virus in the environment to employees, especially when they are removing their PPE.

The studies presented showed the importance of highlighting the survival time of SARS-CoV-2 in the environment, and demonstrated to its relationship with temperature variation and air humidity. The implication of these findings for the pandemic is that the products described here are essential for effective cleaning and disinfection of inanimate areas.

Development of protocols for attending cases of SARS-CoV-2 infection needs to include not only clinical conduct but also use of personal protective equipment for care and cleaning, and disinfection of equipment, surfaces and the environment. The infrastructure for patient and population care is extremely important: it is essential that, in attending these cases, the professionals involved and hence the general population are not exposed to an imminent risk of contamination.

## CONCLUSION

Disinfection of environments, especially those in ordinary use, such as bathrooms, needs to be done constantly. Viral inactivation was seen to occur through using chlorine-based disinfectants, alcohol, detergents, glutaraldehyde, iodine-containing detergents, hydrogen peroxide compounds and household bleaches. Alcohol showed efficient immediate activity. In sewage, sodium hypochlorite was observed to have better action than chlorine dioxide.

## References

[1] Luo C, Yao L, Zhang L (2020). Possible Transmission of Severe Acute Respiratory Syndrome Coronavirus 2 (SARS-CoV-2) in a Public Bath Center in Huai'an, Jiangsu Province, China. JAMA Netw Open.

[2] Fathizadeh H, Maroufi P, Momen-Heravi M (2020). Protection and disinfection policies against SARS-CoV-2 (COVID-19). Infez Med.

[3] To KK, Tsang OT, Chik-Yan Yip C (2020). Consistent detection of 2019 novel coronavirus in saliva. Clin Infect Dis.

[4] Taminato M, Mizusaki-Imoto A, Saconato H (2020). Máscaras de tecido na contenção de gotículas respiratórias − revisão sistemática. Acta Paul Enferm.

[5] Centers for Disease Control and Prevention (CDC) (2020). Coronavirus disease. Considerations for Wearing Masks: Help Slow the Spread of COVID-19. National Center for Immunization and Respiratory Diseases (NCIRD). Division of Viral Diseases.

[6] Kampf G, Todt D, Pfaender S, Steinmann E (2020). Persistence of coronaviruses on inanimate surfaces and their inactivation with biocidal agents. J Hosp Infect.

[7] Rabenau HF, Kampf G, Cinatl J, Doerr HW (2005). Efficacy of various disinfectants against SARS coronavirus. J Hosp Infect.

[8] van Doremalen N, Bushmaker T, Munster VJ (2013). Stability of Middle East respiratory syndrome coronavirus (MERS-CoV) under different environmental conditions. Euro Surveill.

[9] European Centre for Disease Prevention and Control (2020). Disinfection of environments in healthcare and non-healthcare settings potentially contaminated with SARS-CoV-2.

[10] Centers Disease Control and Prevention (2020). Cleaning and Disinfection for Households.

[11] Ouzzani M, Hammady H, Fedorowicz Z, Elmagarmid A (2016). Rayyan-a web and mobile app for systematic reviews. Syst Rev.

[12] Lai MYY, Cheng PKC, Lim WWL (2005). Survival of severe acute respiratory syndrome coronavirus. Clin Infect Dis.

[13] Wang J, Feng H, Zhang S (2020). SARS-CoV-2 RNA detection of hospital isolation wards hygiene monitoring during the Coronavirus Disease 2019 outbreak in a Chinese hospital. Int J Infect Dis.

[14] Wang XW, Li JS, Jin M (2005). Study on the resistance of severe acute respiratory syndrome-associated coronavirus. J Virol Methods.

[15] Booth TF, Kournikakis B, Bastien N (2005). Detection of airborne severe acute respiratory syndrome (SARS) coronavirus and environmental contamination in SARS outbreak units. J Infect Dis.

[16] Sizun J, Yu MW, Talbot PJ (2000). Survival of human coronaviruses 229E and OC43 in suspension and after drying on surfaces: a possible source of hospital-acquired infections. J Hosp Infect.

[17] Walker CM, Ko G (2007). Effect of ultraviolet germicidal irradiation on viral aerosols. Environ Sci Technol.

[18] World Health Organization Coronavirus disease (COVID-19) advice for the public.

[19] World Health Organization (2020). Modes of transmission of virus causing COVID-19: implications for IPC precaution recommendations.

[20] Lénès D, Deboosere N, Ménard-Szczebara F (2010). Assessment of the removal and inactivation of influenza viruses H5N1 and H1N1 by drinking water treatment. Water Res.

[21] Centers for Disease Control and Prevention Cleaning − Guideline for Disinfection and Sterilization in Healthcare Facilities (2008).

[23] Kampf G (2020). Potential role of inanimate surfaces for the spread of coronaviruses and their inactivation with disinfectant agents. Infection Prevention in Practice.

[24] Chia PY, Coleman KK, Tan YK (2020). Detection of air and surface contamination by SARS-CoV-2 in hospital rooms of infected patients. Nat Commun.

[25] Ong SWX, Tan YK, Chia PY (2020). Air, Surface Environmental, and Personal Protective Equipment Contamination by Severe Acute Respiratory Syndrome Coronavirus 2 (SARS-CoV-2) From a Symptomatic Patient. JAMA.

[26] Hota B (2004). Contamination, disinfection, and cross-colonization: are hospital surfaces reservoirs for nosocomial infection?. Clin Infect Dis.

[27] Liu Y, Ning Z, Chen Y (2020). Aerodynamic analysis of SARS-CoV-2 in two Wuhan hospitals. Nature.

[28] Chan KH, Peiris JSM, Lam SY (2011). The effects of temperature and relative humidity on the viability of the SARS coronavirus. Adv Virol.

[29] Centers for Disease Control and Prevention (2020). Interim Infection Prevention and Control Recommendations for Patients with Suspected or Confirmed Coronavirus Disease 2019 (COVID-19) in Healthcare Settings.

